# Bromine-Based Derivatization of Carboxyl-Containing Metabolites for Liquid Chromatography–Trapped Ion Mobility Spectrometry–Mass Spectrometry

**DOI:** 10.1021/jasms.5c00023

**Published:** 2025-03-07

**Authors:** Kaylie I. Kirkwood-Donelson, Prashant Rai, Lalith Perera, Michael B. Fessler, Alan K. Jarmusch

**Affiliations:** † Immunity, Inflammation, and Disease Laboratory, National Institute of Environmental Health Sciences, National Institutes of Health, Research Triangle Park, North Carolina 27709, United States; ‡ Genome Integrity and Structural Biology Laboratory, National Institute of Environmental Health Sciences, National Institutes of Health, Research Triangle Park, North Carolina 27709, United States

## Abstract

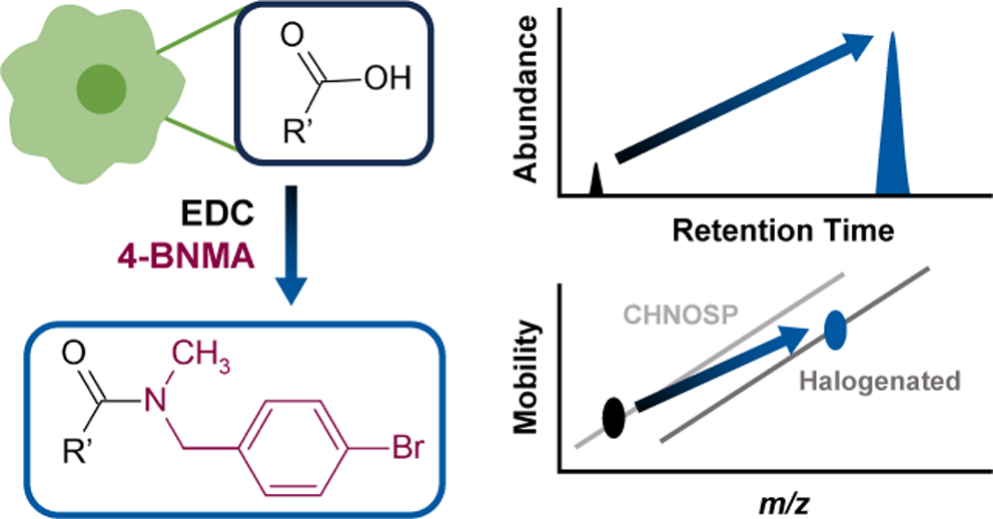

The analysis of small carboxyl-containing metabolites (CCMs), such as tricarboxylic acid (TCA) cycle intermediates, provides highly useful information about the metabolic state of cells. However, their detection using liquid chromatography–electrospray ionization–tandem mass spectrometry (LC-ESI-MS/MS) methods can face sensitivity and specificity challenges given their low ionization efficiency and the presence of isomers. Ion mobility spectrometry (IMS), such as trapped ion mobility spectrometry (TIMS), provides additional specificity, but further signal loss can occur during the mobility separation process. We, therefore, developed a solution to boost CCM ionization and chromatographic separation as well as leverage specificity of IMS. Inspired by carbodiimide-mediated coupling of carboxylic acids with 4-bromo-*N*-methylbenzylamine (4-BNMA) for quantitative analysis, we newly report the benefits of this reagent for TIMS-based measurement. We observed a pronounced (orders of magnitude) increase in signal and enhanced isomer separations, particularly by LC. We found that utilization of a brominated reagent, such as 4-BNMA, offered unique benefits for untargeted CCM measurement. Derivatized CCMs displayed shifted mobility out of the metabolite and lipid region of the TIMS-MS space as well as characteristic isotope patterns, which were leveraged for data mining with Mass Spectrometry Query Language (MassQL) and indication of the number of carboxyl groups. The utility of our LC-ESI-TIMS-MS/MS method with 4-BMA derivatization was demonstrated via the characterization of alterations in CCM expression in bone marrow-derived macrophages upon activation with lipopolysaccharide. While metabolic reprogramming in activated macrophages has been characterized previously, especially with respect to TCA cycle intermediates, we report a novel finding that isomeric itaconic, mesaconic, and citraconic acid increase after 24 h, indicating possible roles in the inflammatory response.

## Introduction

Analysis of metabolites and other small molecules containing a carboxyl group (e.g., amino acids, fatty acids, and tricarboxylic acid (TCA) cycle intermediates), referred to here as carboxyl-containing metabolites (CCMs), is imperative given their critical biochemical functions and wide distribution. For example, TCA cycle intermediates are small, polar CCMs that have central roles in energy metabolism and cellular signaling processes, among others.^1,2^ Measurement of CCMs can be achieved using gas chromatography–mass spectrometry (GC-MS) following silylation.^3−5^ But liquid chromatography–electrospray ionization–tandem mass spectrometry (LC-ESI-MS/MS) is more common for untargeted metabolomics due to the less harsh ionization conditions and lesser requirements for sample preparation. CCM analysis by LC-MS can be complicated by poor retention and low ionization efficiency when using reversed-phase LC (RPLC) and negative ESI (ESI^–^) mode methods. Alternative LC approaches using hydrophilic interaction chromatography (HILIC),^6−8^ ion pairing,^9−11^ or ion exchange/exclusion chromatography^12−14^ have been employed; however, these approaches do not address the low sensitivity in ESI^–^ mode and, in some cases, lead to further ionization suppression.^15^

When more comprehensive characterization or further separation of metabolites is warranted, researchers have employed ion mobility spectrometry (IMS) coupled to LC-MS. IMS is a gas-phase separation technique in which ions are differentiated by their size, shape, and charge. In trapped IMS (TIMS), an electric field is applied to an augmented ion funnel to trap ions against the carrier gas. The electric field gradient is gradually reduced to elute ions with ascending mobilities, i.e., largest to smallest size.^16^ IMS provides numerous benefits without increasing analysis time, as it is rapid enough to be nested between LC separation and MS measurements.^17,18^ The observed ion mobility can be related to its ion-neutral collision cross section (CCS) value, facilitating more accurate identifications and chemical structure assignments.^19^ TIMS offers a unique scan mode, parallel accumulation serial fragmentation (PASEF), which synchronizes TIMS separations with the MS/MS precursor selection. Utilizing PASEF allows for fragmentation of multiple precursors in a single TIMS scan, thereby increasing MS/MS acquisition rates while maintaining sensitivity and preventing coselection of unwanted isobaric precursor ions.^20,21^ While IMS can aid in overcoming some of the challenges associated with analyzing CCMs, such as separation of isobaric or isomeric species (e.g., citric and isocitric acid)^22,23^ and MS/MS selection of low-abundance precursors, it is not without drawbacks. Very small (<200 Da) ions, including some CCMs, are subject to transmission loss during the TIMS process due to the longer trapping times and RF bias.^16^ Researchers seeking comprehensive metabolomics data with this instrumentation may use multiple injections, one with TIMS mode on to make use of the aforementioned benefits of TIMS, and one with TIMS mode off to capture CCMs and other small metabolites that may be lost during separation.^24,25^

**Scheme 1:**
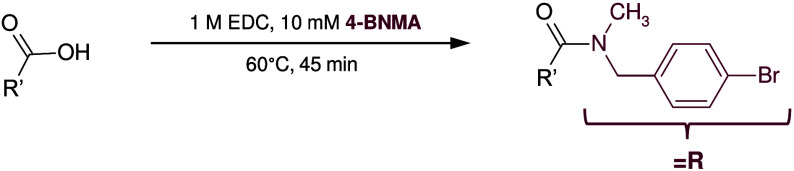
Overview of Carbodiimide-Mediated Derivatization of Metabolites Containing a Carboxyl Group with 4-BNMA

Chemical derivatization prior to GC or LC-MS analysis has many advantages for increasing sensitivity for specificity. Notably, carboxylic acids can be modified using different derivatization approaches including acyl halides and carbodiimide-mediated amide coupling to add distinctive mass, isotope patterns, improve ionization, alter retention times, etc.^26−33^ Marquis et al. demonstrated the utility of 1-ethyl-3-dimethylaminopropyl carbodiimide (EDC)-mediated coupling of TCA intermediates with 4-bromo-*N*-methylbenzylamine (4-BNMA) for a targeted, quantitative approach.^27^ In their objective of improving quantitative measurement, they found a substantial improvement to signal and chromatographic separation. Notably, derivatization with 4-BNMA facilitated a switch of the measurement polarity from ESI^–^ to ESI^+^ and separation of the isomers citric acid and isocitric acid. Marquis et al. reported limits of detection ranging from 0.2 to 44 ng/mL for 4-BNMA derivatized TCA intermediates using a Qtrap-based multiple reaction monitoring (MRM) method.^27^Scheme 1 gives an overview of the optimized derivatization procedure presented by Marquis et al., which can be performed under mild, aqueous conditions using commercially available and inexpensive reagents.^27^

Chemical derivatization can also be leveraged to improve IMS separations. For example, IMS shift reagents selectively shift the gas-phase mobility of an analyte or increase the resolution of isomers through covalent or noncovalent interactions via chemical derivatization,^34^ formation of adducts or clusters including metal cations,^35−38^ or polar buffer gas additives (i.e., dopants or chemical modifiers) such as primary and secondary alcohols.^39−41^ Biomolecular classes have distinct mobility–mass correlations due to their unique gas-phase packing efficiencies,^42,43^ resulting in signal occupying only a portion of the possible IMS-MS space. Therefore, substantial mobility shifts can be applied to shift analytes containing specific chemical functionalities to previously unoccupied IMS-MS regions, providing separation from analytes of similar mass and decreased background noise in complex mixtures. This concept was initially applied to extract phosphorylated peptides from the expected peptide IMS-MS region using crown ethers.^44^ Bulky crown ethers decrease the mobility, or increase the CCS, of ions more than they increase the mass, leading to signals above the peptide mobility–mass trendline. Conversely, shift reagents that shift signals below the expected IMS-MS region for a given class of compounds typically include lanthanide metals or halogens, which have been shown to increase the mass of ions more than they decrease their mobility.^45−47^ For example, Hynds and Hines leveraged halogenated Paternò–Büchi reagents to characterize lipid carbon–carbon double bond positions while shifting the PB derivatized lipids outside of the lipid IMS-MS space,^34^ and Kerr et al. used various lanthanide metal chelating agents for multiplexed characterization of peptide functionalities.^47^ We recognized the potential of 4-BNMA as a mobility shift reagent for CCMs to decrease noise, ensure PASEF MS/MS selection, and aid in analyte classification.

Inspired by the derivatization approach from Marquis et al. for quantitative analysis, we developed and report a workflow for CCM characterization encompassing derivatization, LC-TIMS-MS/MS measurement, and data analysis. 4-BNMA was chosen as the derivatization reagent for its potential to act as a brominated IMS shift reagent, enhance the signal, and improve LC separation. We confirmed improved ionization and LC separation and report novel IMS measurements and benefits using authentic chemical standards. To demonstrate use in metabolomics applications, we applied our derivatization and untargeted LC-TIMS-MS/MS method to profile CCM changes following lipopolysaccharide (LPS) activation of murine bone marrow-derived macrophages (BMDMs), an exposure that is known to incite metabolic reprogramming. In doing so, we recapitulated previously reported changes in TCA cycle CCMs and itaconic acid while observing novel increases in the stereoisomers citraconic and mesaconic acid after 24 h.

## Methods

### Materials

Chemical standards for pyruvic acid (cat. no. 107360), citric acid (cat. no. C0759), itaconic acid (cat. no. I29204), citraconic acid (cat. no. C82604), and mesaconic acid (cat. no. 131040) were obtained from Sigma-Aldrich (St. Louis, MO, USA). The chemical standard for isocitric acid (cat. no. 30378) was obtained from Cayman Chemical Company (Ann Arbor, MI, USA). Chemical standards were prepared at an initial concentration of 1 mg/mL in water and subsequently diluted with water. Derivatization reagents 4-bromo-*N*-methylbenzylamine (4-BNMA, cat. no. 631140) and *N*-(3-dimethylaminopropyl)-*N*′-ethylcarbodiimide hydrochloride (EDC, cat. no. E7750) were obtained from Sigma-Aldrich (St. Louis, MO, USA). 10 mM 4-BNMA was prepared in acetonitrile, and 1 M EDC was prepared in acetonitrile/water (9:1) *v/v*. Fetal bovine serum (FBS, cat. no. S11150H) was obtained from R&D Systems. Penicillin/streptomycin and lipopolysaccharide (LPS) were obtained from Sigma-Aldrich. Reconstitution, extraction, and mobile phase solvents (water, methanol, acetonitrile) were Optima LC-MS grade from Fisher Scientific (Hampton, NH, USA). LiChropur acetic acid was obtained from Supelco (Bellefonte, PA, USA).

### BMDM Sample Preparation

Bone marrow cells were isolated from the tibia and femur of C57/BL6 mice (The Jackson Laboratory, Bar Harbor, ME, USA) and grown in L929-supernatant (10%) enriched complete DMEM medium (10% FBS and penicillin/streptomycin) for 6 days to differentiate them to macrophages. On day 6, BMDMs were seeded in complete DMEM medium in 6-well plates at 2.5 × 10^6^ cells per well and allowed to rest overnight. LPS (*E. coli* O111:B4) was used at 100 ng/mL. At 4 and 24 h, cells were washed with PBS twice, collected by scraping, and spun down. 1 mL of prechilled (−80 °C) extraction solvent of methanol/water (4:1) *v/v* was added to each tube, vortexed for 10 s, and stored at −20 °C for 30 min. Cell lysates were centrifuged at 14 000*g* for 10 min at 4 °C. 900 μL of supernatant was transferred to a new tube and stored at −80 °C, then dried via centrifugal evaporation. Dried cell lysates were stored at −80 °C until derivatization.

### Derivatization Procedure

The derivatization procedure carried out here was adapted from the optimized method previously described by Marquis et al.^27^ For derivatization of chemical standards, 15 μL of organic acid standards at known concentrations in water was transferred to a microcentrifuge tube. For quantitative assessment of itaconic acid and its isomers, 15 μL mixtures of all three standards were prepared at starting concentrations ranging from 17 ng/mL to 170 μg/mL. For derivatization of BMDMs, reagents were added directly to the dried cell lysates. Method blanks were prepared by using 15 μL of water. EDC (25 μL at 1 M) and 4-BNMA (50 μL at 10 mM) were added to each sample, standard, or method blank and allowed to react for 45 min at 60 °C using a block heater. The reaction mixtures were then dried via centrifugal evaporation and reconstituted with 80 μL of acetonitrile/water (1:1) *v/v*, centrifuged for 10 min at 14 000 rcf and 4 °C, and transferred to glass vials.

### Instrumentation

Samples analyses were carried out on a platform coupling high performance liquid chromatography (Elute LC, Bruker Daltonics, Bremen, Germany) with trapped ion mobility spectrometry–quadrupole time-of-flight mass spectrometry (timsTOF Pro, Bruker Daltonics). For HPLC separation, 5 μL of sample was injected onto a Kinetex C18 column (2.1 mm × 100 mm, 2.6 μm) equipped with an HPLC guard cartridge and held at 30 °C (Phenomenex, Torrance, CA, USA). Gradient elution was performed at a flow rate of 0.5 mL/min using water with 0.1% acetic acid (mobile phase A) and acetonitrile with 0.1% acetic acid (mobile phase B). Separation was performed as follows: 10% B from 0 to 0.75 min, 10% to 95% B from 0.75 to 12 min, 95% B from 12 to 14 min, 95% to 10% B from 14 to 15.5 min, and 10% B from 15.5 to 16.5 min. During the first 0.3 min of each run, a mixture of ESI-L Low Concentration Tuning Mix (cat. no. G1969-85000, Agilent Technologies, Santa Clara, CA, USA) and sodium formate (9:1) *v/v* was directly infused for internal mobility and mass calibration. In addition to the online internal calibration, TIMS and TOF were calibrated prior to data acquisition using the same tuning mix/sodium formate solution.

Data were collected in positive ionization mode with ESI source parameters as follows: 10 L/min drying gas at 220 °C, 4500 V capillary voltage, −500 V end plate offset, and 2.2 bar nebulizer pressure. A mass scan range of *m*/*z* 50–1300 was set. The TIMS device was operated in custom mode over an inverse reduced mobility (1/*K*_0_) range of 0.4–1.5 V·s/cm^2^ with a ramp time of 100 ms (9.43 Hz), which included one full TIMS-MS scan and two PASEF MS/MS scans. Collision energy was applied at 20 eV, and PASEF active exclusion was employed with an exclusion release time of 0.1 min. The TIMS funnel RF potential was set to 300 Vpp. An ion charge control target intensity of 7.5 million was set to prevent TIMS funnel saturation and space charge effects.

### Data Analysis

The mass and mobility values were automatically calibrated post hoc on a file-by-file basis using reference standards of known mass, mobility, and CCS,^19,48^ which were directly infused for the first 0.3 min of each sample run. Data files were initially inspected using Data Analysis version 5.3.236 (Bruker Daltonics). To evaluate metabolites with derivatized reference standards, raw (.d) data files were imported into a Skyline (v23.1.0.455)^49^ document containing the name, molecular formula and adduct (thereby theoretical *m*/*z*), retention time, and CCS values gathered from the reference standards. MS and ion mobility filters were applied at resolving powers of 50 000 and 60, respectively. Conservative values were applied to ensure all signal was captured. Identifications were confirmed using the following criteria: mass error < 5 ppm, isotope dot product (idotp) > 0.9, retention time alignment within ±0.1 min, and calculated CCS within 1% of the reference standard. Additionally, PASEF MS/MS data were matched to the reference standards. External calibration curves were generated in Skyline for itaconic acid, citraconic acid, and mesaconic acid using linear regression with no weighting. The mobility filtering window was widened to capture both conformers for itaconic acid. 170 μg/mL standards were above the linear range and were excluded for curve generation. Extracted chromatographic peak areas or calculated concentrations were exported to Excel for further analysis.

For untargeted profiling of all derivatized carboxylic acids, raw (.d) data files were first converted to mzML files using TIMSCONVERT;^50^ then, MassQL (v31.4)^51^ was employed to pinpoint all features that possessed characteristic bromine isotope distributions and the diagnostic *m*/*z* 168.965 fragment ion. The resulting *m*/*z* and retention time values were first filtered for duplicates and then imported into Skyline. Additional feature filtering was based on the absence of signal in the processing blanks and abundance. Rather than a set intensity threshold, features were retained at this stage only if the entire isotope distribution was captured in order to confirm the number of bromine atoms. Finally, redundant features, i.e., dimers, in-source fragments, or various adducts of the same molecule, were filtered out. CCS values were calculated by Skyline for all remaining unknown features. Then, mobility filtering was applied with a resolving power of 60. For tentatively identified unknown features, observed *m*/*z* values from MassQL were replaced with the molecular formula and adduct to evaluate the mass error and idotp, in addition to the PASEF MS/MS data. Extracted peak areas were exported to Excel for further analysis. Statistical analyses were performed using R via Jupyter Notebook. Fold change (FC) was calculated for each pairwise comparison, and *p*-values were calculated using one-way analysis of variance (ANOVA) followed by a post hoc Tukey’s Honest Significant Difference (HSD) test to adjust for multiple hypothesis testing.

### Energy Optimization

For all compounds investigated, all possible conformations were generated and clustered using the CCDC module Mogul. These conformations were energy minimized using Gaussian-16 first at the semiempirical PM6 level. The top 100 lowest energy conformations of each system were further energy minimized by using Gaussian-16 at the B3LYP/6-31g(d,p) level.

## Results and Discussion

### Derivatization with 4-BNMA Increased Signal of CCMs

**Figure 1:**
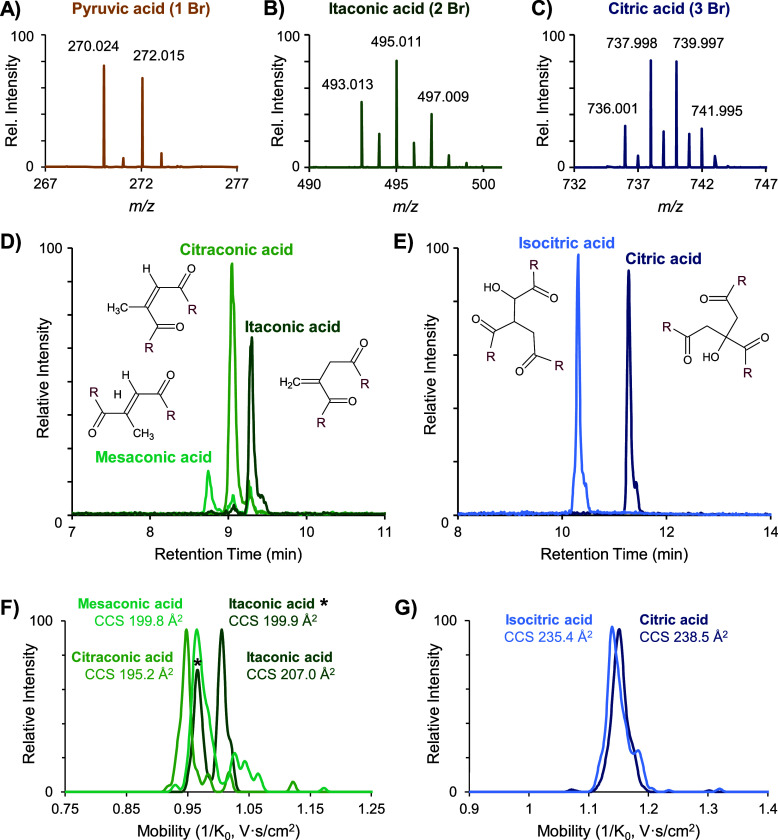
Spectral characterization of 4-BNMA derivatized carboxylic acid standards. Mass spectra displaying example isotope distributions for (A) pyruvic acid (monocarboxylic acid), (B) itaconic acid (dicarboxylic acid), and (C) citric acid (tricarboxylic acid). Overlaid extracted ion chromatograms for 4-BNMA derivatized isomers (D) itaconic, citraconic, and mesaconic acid (*m*/*z* 493.012) and (E) citric and isocitric acid (*m*/*z* 736.002) with corresponding chemical structures, where R = 4-BNMA. Overlaid extracted ion mobilograms for 4-BNMA derivatized (F) itaconic, citraconic, and mesaconic acid and (G) citric and isocitric acid.

Pyruvic acid was analyzed by LC-TIMS-MS/MS to determine the improvement in signal upon derivatization with 4-BNMA. Nonderivatized pyruvic acid was not detected in ESI^–^ mode at concentrations below 10 μg/mL. At this concentration, it was only detected as a low-abundance dimer, whereas 4-BNMA derivatized pyruvic acid was detected as a protonated ion in ESI^+^ mode at concentrations as low as 50 ng/mL, that is, approximately 3 orders of magnitude lower. The improvement in signal likely translates to an improvement in sensitivity following derivatization; however, determination of the limit of detection was not performed, as quantitative analysis was not a primary objective for the LC-TIMS-MS/MS-based characterization. Marquis et al. previously characterized limits of detection for 4-BNMA derivatized TCA metabolites using a method more well-suited for quantitative analysis with a triple quadrupole instrument and mass labeled internal standards to produce absolute concentrations.^27^ The observed signal increase is proposed to be multifactorial but almost certainly results from improved gas-phase basicity and hydrophobicity as well as increased mass and size. Unmodified, it is favorable to detect CCMs via ESI^–^, requiring the analyte to be deprotonated at a carboxyl group. Further, CCMs can be polyprotic and thus exist in multiple states of deprotonation. Modified by 4-BNMA, analytes can be detected in ESI^+^, exploiting the gas-phase basicity of a tertiary amine.^26^ The addition of a phenyl group via 4-BNMA increases hydrophobicity and concurrently increases the surface activity of the analyte during ESI^+^, which also leads to more effective ionization.^52,53^ These factors are likely as equally applicable to our analysis as they were in Marquis et al. Another important factor in the signal increase, a unique observation related to our experiment, was the increased mass and size of 4-BNMA derivatives, as signal loss can occur within the TIMS device for very small ions (<200 Da). Ions of larger size (decreased mobility) are trapped in the TIMS device for less time than smaller ions and better confined in the radial direction to prevent diffusion and neutralization.^54^ All these factors combined to yield the illustrative example of pyruvic acid, which increased from a nonderivatized [M – H]^−^ of *m*/*z* 87.008 to a derivatized [M + H]^+^ of *m*/*z* 270.012 (Figure 1A, Table S1).

### Mass Spectral Observations of 4-BNMA Derivatized CCMs

Derivatization with 4-BNMA improved the selectivity of CCMs by increasing analyte *m*/*z* (Table S1) and altering the isotopic distribution of each ion given the unique stable isotope composition of bromine (50.7% ^79^Br and 49.3% ^81^Br). This provided a unique ability to determine any CCMs modified by 4-BNMA as well as the number of carboxylic acid groups in CCMs. Figure 1A–C depicts the [M + H]^+^ isotope patterns for the illustrative mono-, di-, and triderivatized standards pyruvic acid, itaconic acid, and citric acid, respectively. We assessed the reaction completion by our LC-IMS-MS method and observed simple mass spectra dominated by the predicted singly charged *m*/*z* values. Mono-, di-, or tricarboxylic acids had one *m*/*z* corresponding with the predicted one, two, or three 4-BNMA additions, respectively. For example, we did not detect *m*/*z* values of mono- or doubly derivatized citric acid. By nature of our assessment, we cannot rule out incomplete reactions which, given our primary aim of untargeted metabolomics and not quantitative analysis, are outside of the scope.

### Liquid Chromatography Separations of 4-BNMA Derivatized CCMs

It has been demonstrated that derivatization of carboxylic acids with 4-BNMA and other phenyl-containing reagents greatly increases their retention in reversed-phase LC.^26,27^ This was confirmed here, where measured RPLC retention times ranged from 0.6 to 1.1 min for nonderivatized standards and from 5.4 to 11.2 min for derivatized standards using a 16.5 min gradient method (Table S1). Derivatization with 4-BNMA led to improved isomer separations. Figure 1D,E highlights the extracted ion chromatograms (EICs) of two key sets of 4-BNMA derivatized CCM isomers, which are well-separated by RPLC. The dicarboxylic acid itaconic acid and its constitutional isomers mesaconic and citraconic acid were of particular interest for the macrophage activation application discussed below; thus, the ability to distinguish them was crucial. Near-baseline separation was achieved for all three 4-BNMA derivatized isomers (Figure 1D). This was most notable for mesaconic and citraconic acid, which are more structurally similar as *cis*/*trans* stereoisomers and coelute at 0.7 min when unmodified. Impurities were present in the neat chemical standards (reported as 98–99% pure), also noted by Chen et al.,^55^ giving rise to minor signals at the retention times of the other two isomers. The TCA cycle intermediates citric and isocitric acid are constitutional isomers, differing by the position of a hydroxyl group. Once derivatized, this isomer pair was well-resolved with nearly 1 min of separation (Figure 1E), akin to the results obtained by Marquis et al.^27^ To further investigate our empirical data, we performed modeling on the two sets of derivatized CCM isomers. Relative dipole moments of the resulting energy-optimized structures support their relative polarity and elution order, with average dipole moments of isocitric acid > citric acid and mesaconic > citraconic > itaconic acid.

### Ion Mobility Separations of 4-BNMA Derivatized CCMs

The impact of 4-BNMA derivatization on IMS isomer separations was evaluated by using the same isomers evaluated above for RPLC separations (Figure 1F,G). The first set of constitutional isomers comprising itaconic, citraconic, and mesaconic acid are a notable challenge for IMS given that citraconic and mesaconic acid are *cis*/*trans* isomers. The anticipated percent difference in CCS value between constitutional isomers is <3% and less than ∼1% for *cis*/*trans* isomers.^56^ The 4-BNMA derivatized *cis*/*trans* isomers citraconic and mesaconic acid had a larger CCS difference than expected, ΔCCS of 2.3%, yet they were not well-resolved at the ∼60 resolving power (*R*_p_) used in our untargeted metabolomics TIMS measurements. The measured CCS values were 195.2 and 199.8 Å^2^ for citraconic acid and mesaconic acid, respectively (Figure 1F). However, near-baseline separation was achieved between citraconic acid and itaconic acid (207.0 Å^2^), ΔCCS = 5.9%, and between mesaconic acid and itaconic acid, ΔCCS = 3.5%. Empirical CCS values for all three underivatized isomers were not available, and thus, improvement to isomer separation is difficult to judge. CCS base predictions based on the isomer SMILES structures indicate that far less than 1% difference in CCS is likely (Table S1).^57^

Interestingly, derivatized itaconic acid had two distinct mobility distributions with 1/*K*_0_ values of 1.01 (major) and 0.97 (minor) V·s/cm^2^ (Figure S1). The mobility of the minor signal was aligned with mesaconic acid (Figure 1F and Figure S1, CCS = 199.9 and 199.8 Å^2^), suggesting derivatized itaconic acid undergoes gas-phase isomerization into derivatized mesaconic acid. This split distribution was characteristic of itaconic acid, as it was also observed when derivatized itaconic acid was detected as other adducts ([M + Na]^+^, [2M + H]^+^). Modeling of the energy-optimized structures (Figure S2) supports the possible conversion of itaconic acid to mesaconic acid, with an energy activation barrier within the typical range of internal energy deposition during ESI (<3 eV). Resonance stabilization and the electronegativity of bromine may have aided in the proton transfer required for isomerization. Further investigation would be needed to discern whether this gas-phase isomerization occurs with nonderivatized itaconic acid or only 4-BNMA derivatized itaconic acid, as no studies characterizing the mobility of these isomers (beyond reporting the [M – H]^−^ CCS values) could be found. Itaconic acid’s IMS observation does not compromise distinguishing 4-BNMA derivatized itaconic or mesaconic acid as they are chromatographically separated, but we thought it remarkable enough to explore and provide rationale.

The 4-BNMA derivatized tricarboxylic acids isocitric and citric acid had empirical CCS values of 235.4 and 238.5 Å^2^, giving a ΔCCS of 1.3% (Figure 1G). The percent difference in CCS value matches closely with the expectation for *cis*/*trans* isomers. The minimum energy ion structures aligned with the observed relative mobility and CCS values, where derivatized isocitric acid has a more compact structure than citric acid (Figure S3). As to any improvement in isomer resolution, Nichols et al. reported nonderivatized [M + Na]^+^ CCS values of 142.7 and 143.1 Å^2^ for isocitric and citric acid (ΔCCS < 0.3%), respectively, with even less mobility separation in ESI^–^ mode ([M – H]^−^ CCS values of 127.0 and 127.1 Å^2^).^22^ These isomers would be challenging to separate even with the highest *R*_p_ instrumentation, such as structures for lossless ion manipulations (SLIM), which can achieve an *R*_p_ of 300, requiring a ∼0.6% CCS difference for near-baseline resolution.^58^ The TIMS instrumentation utilized here is also capable of high-resolution ion mobility separations (*R*_p_ ∼ 200 for singly charged ions);^54^ however, the ultraresolution operating mode is not well-suited for untargeted measurements on the LC time scale.^16^ Given the ΔCCS of each isomer pair investigated here (1.3–5.9%), full resolution of the 4-BNMA derivatized isomers of interest should be readily attainable with higher-resolution ion mobility approaches.^54,58−61^

### IMS-MS Observations of 4-BNMA Derivatized CCMs

**Figure 2:**
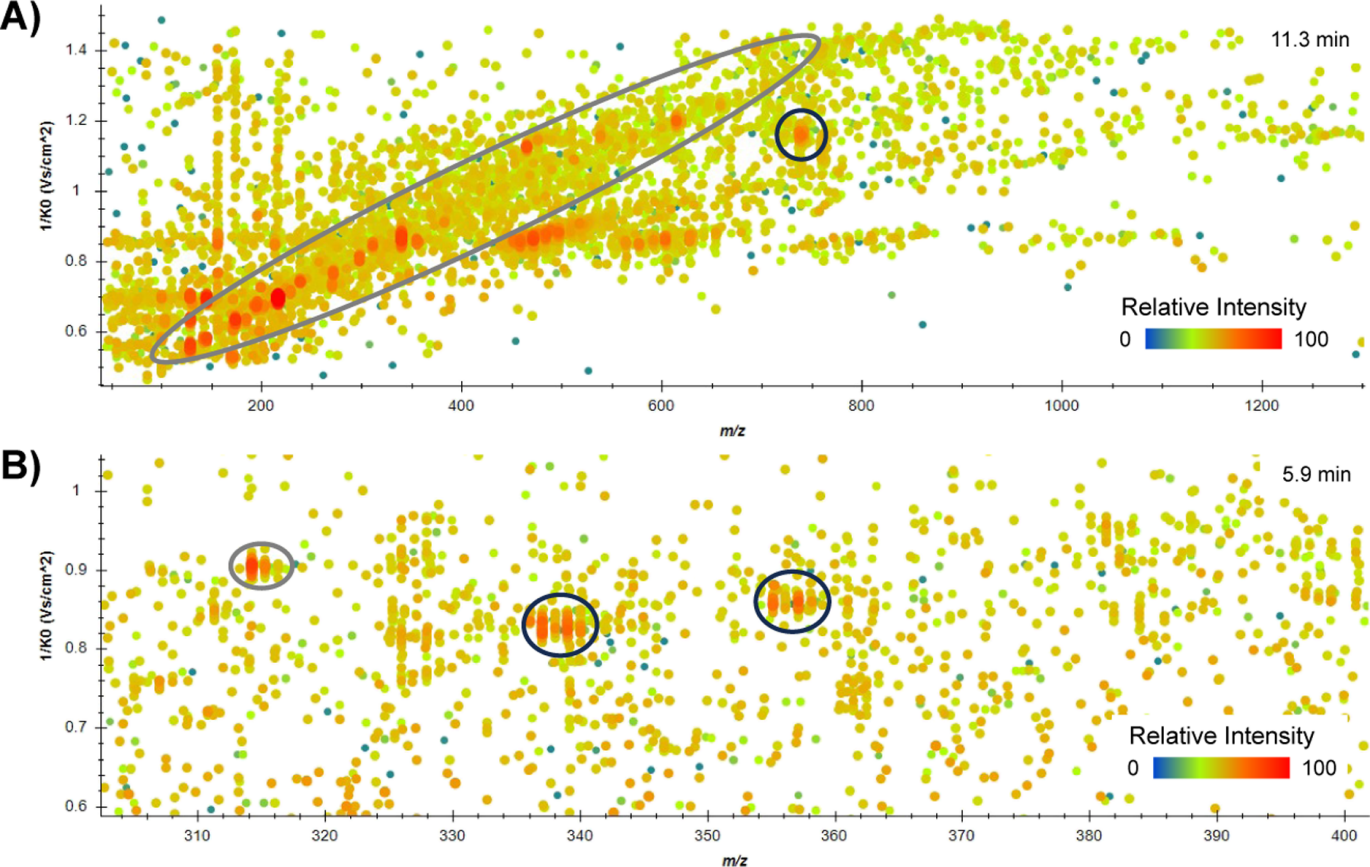
IMS-MS heatmaps of 4-BNMA derivatized CCMs in complex matrix. Nonhalogenated metabolite and lipid feature space is approximated with gray dashed circles, and halogenated features are outlined with black solid circles. (A) Full IMS-MS heatmap at 11.3 min showing the shifted inverse reduced mobility (1/*K*_0_) of 4-BNMA derivatized citric acid compared to the general nonhalogenated space. (B) Zoomed-in IMS-MS heatmap at 5.9 min showing two monobrominated features compared to a nonhalogenated feature at a similar (lower) mass but higher inverse reduced mobility.

Given the vast LC separation of isomers and modest improvement to gas-phase isomer separation, the primary benefit of adding the TIMS dimension to this workflow is the mobility-based separation of derivatized analytes from nonhalogenated signals. As expected, the derivatized carboxylic acids were shifted considerably in mass and size (mobility or CCS). For example, the tricarboxylic acid citric acid was large enough to be detected with TIMS mode on as a deprotonated ion, albeit at low abundance, and had an *m*/*z* of 191.019 and a CCS of 128.4 Å^2^, in agreement with the literature value of 129.5 Å^2^ (0.9% difference) from a DTIMS platform.^43^ Following derivatization with 4-BNMA, citric acid was detected as a protonated ion at an *m*/*z* of 736.002 and CCS of 238.5 Å^2^, or a 117% increase in molecular weight and 60% increase in CCS. Halogens are known to disproportionately increase the mass of a compound relative to its size, an effect that becomes more pronounced with the addition of more bromine atoms on derivatized di- and tricarboxylic acids.^45,46^ This is demonstrated in Figure S4, where the slope of each line connecting the CCS versus the *m*/*z* point before and after derivatization decreases with the addition of each bromine, confirming that more halogenation led to a more pronounced shift in mobility. Figure 2 shows example IMS-MS heatmaps, where the mobilities of derivatized ions were sufficiently shifted below metabolite/lipid (nonhalogenated) space. Therefore, 4-BNMA is an ion mobility shift reagent via covalent bonding. Leveraging the mobility shift in the form of signal filtering can reduce noise.^62^ An example is shown in Figure S5, where signal from a nonhalogenated ion of higher relative abundance than a dibrominated ion with an overlapping isotopic distribution is removed when a mobility filter is imposed. Moreover, as PASEF selection is based on the mobility of an ion relative to the other ions present in that scan,^20^ shifting the mobility of derivatized ions likely increased the number of carboxylic acids selected for MS/MS.

### Untargeted Metabolomics Measurement of CCMs in Activated Bone Marrow-Derived Macrophages

The derivatization and untargeted LC-TIMS-MS/MS approach was applied to bone marrow-derived macrophage (BMDM) samples to evaluate changes in CCM expression following lipopolysaccharide (LPS) stimulation, which activates the macrophage’s inflammatory response. Macrophages are important innate immune cells which play a crucial role in pro-inflammatory responses to combat infections as well as immunomodulation to resolve inflammation.^63^

One notable difference between the analysis of standards and biological samples was the observation of partial derivatization in the complex biological matrix. However, the partially derivatized products, i.e. tricarboxylic acid with only two 4-BNMA modifications, were at relatively low abundances (<10% of fully derivatized peak areas) and shared the same trends as the fully derivatized products (Figure S6). That is, we do not anticipate that incomplete derivatization would impact the interpretation of the following untargeted metabolomics results, and partially derivatized products were disregarded.

**Figure 3:**
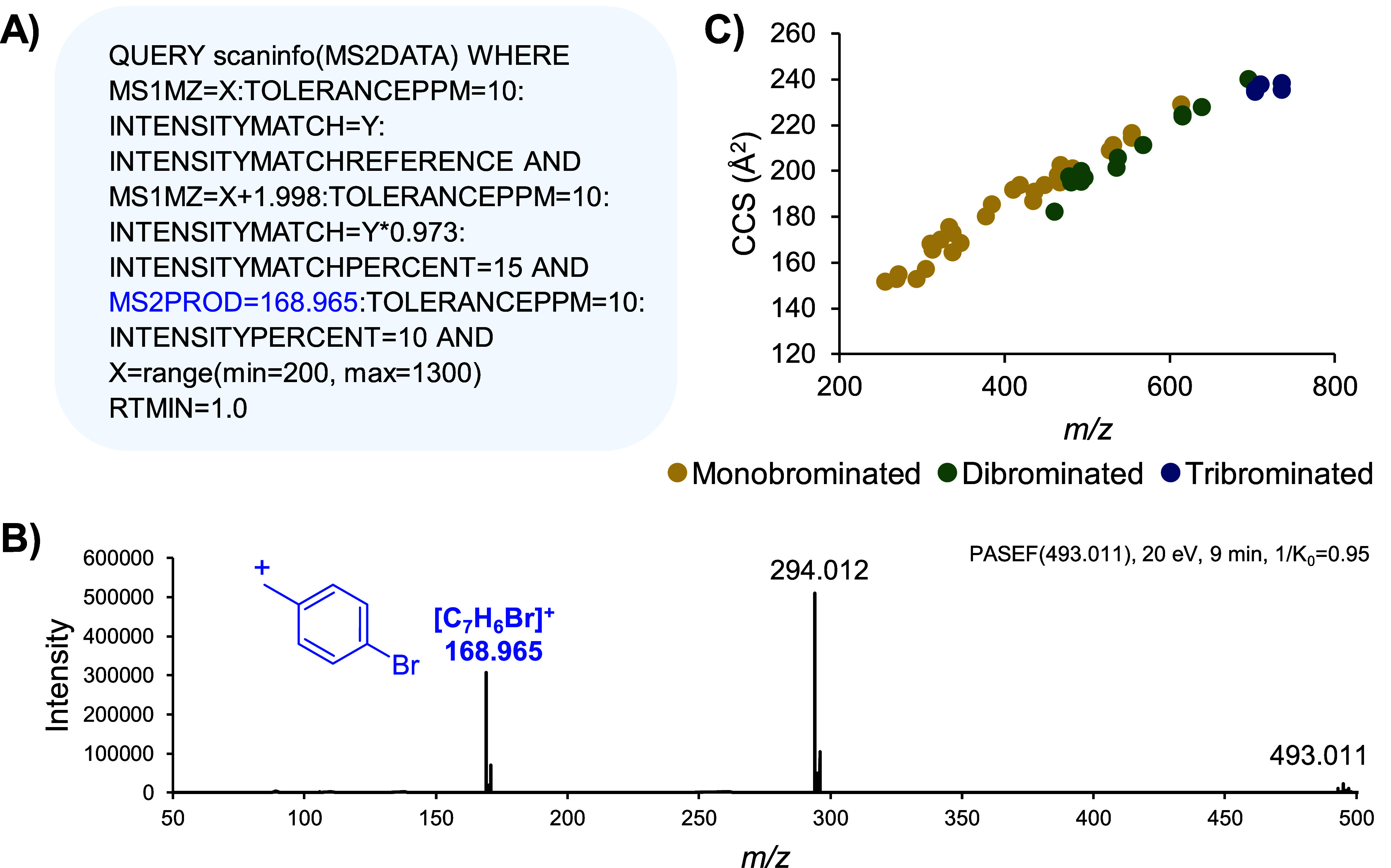
Untargeted BMDM 4-BNMA derivatized CCM analysis. (A) MassQL query applied to search data files for all features between *m*/*z* 200 and 1300 and a minimum retention time of 1.0 min with the expected isotope pattern (precursor *m*/*z* of X at intensity Y and M + 2 (X + 1.998) at intensity Y × 0.973 with 10 ppm mass tolerance and 15% intensity match tolerance) and fragment ion (*m*/*z* 168.965 with 10 ppm mass tolerance at ≥10% relative intensity). (B) Example PASEF MS/MS spectrum of 4-BNMA derivatized citraconic acid displaying the expected *m*/*z* 168.965 product ion. (C) IMS-MS plot for all unique derivatized carboxylic acids detected in BMDM samples, colored by the number of bromine atoms and therefore the number of carboxyl groups.

Mass Spectrometry Query Language (MassQL)^51^ was employed to search the raw data for all spectral features with the characteristic isotopic distribution and fragment ion expected of derivatized CCMs. The MassQL query used for monobrominated features is shown in Figure 3A, which flags all features with the approximate isotope pattern in Figure 1A and the 4-bromophenylmethylium fragment of *m*/*z* 168.965 (Figure 3B). This software approach is unique in that it parses the data files without the need for feature finding or data processing *a priori*. Nearly 400 signals were extracted using MassQL, which were rigorously filtered for false positives, repetitive entries, noise, presence in processing blanks, and redundancy (dimers, in-source fragments, other adducts besides [M + H]^+^, or partially derivatized forms of the same molecule). Strict post hoc filtering was performed to minimize false discoveries at the expense of a comprehensive investigation, and it is likely that more derivatized CCMs could be detected. We focused on 50 unique putative derivatized CCMs after manually validating the number of derivatized acidic sites via an isotope pattern, although many more were detected but impractical to evaluate. The calculated CCS and observed *m*/*z* values of the 50 derivatized CCMs are plotted in Figure 3C, which further demonstrates the increase in mobility shift with increasing halogenation. Of the 50 unique CCMs detected in the BMDM samples, 28 were monocarboxylic acids, 16 were dicarboxylic acids, and 6 were tricarboxylic acids. Upon further manual interpretation, the derivatized CCMs were putatively assigned annotations (Table S2). Notably, our approach was suitable to detect TCA cycle organic acid intermediates (e.g., citric acid, succinic acid, fumaric acid), acidic amino acids and their derivatives (e.g., aspartic acid, glutamic acid, *N*-acetylaspartic acid), and other small CCMs (e.g., glutaric acid, acetylcarnitine, gluconic acid). MassQL and the distilled information were used in the discovery phase of our interpretation and informed subsequent analysis.

**Figure 4:**
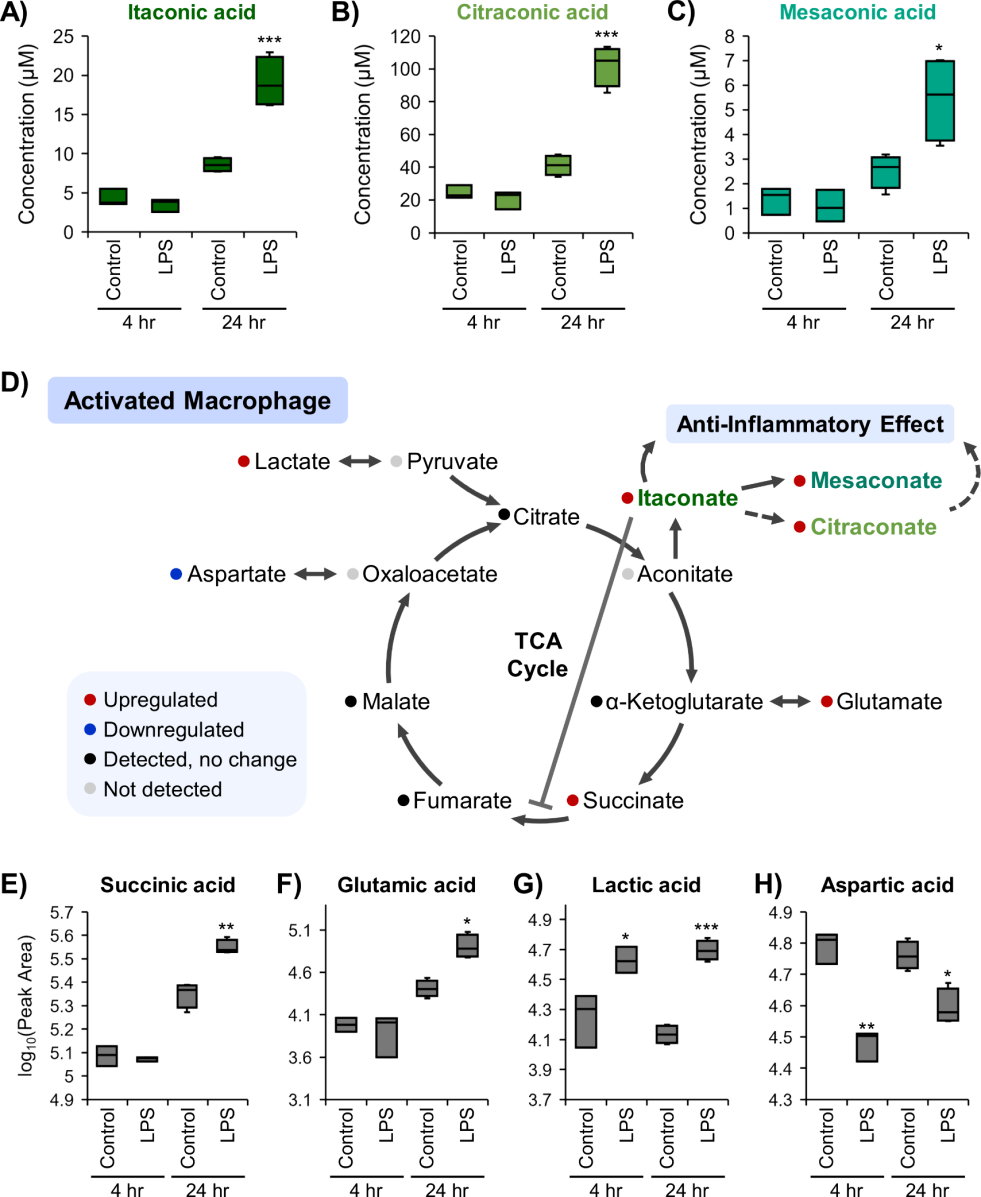
TCA cycle intermediate and related metabolite expression following macrophage activation. Calculated concentrations of the isomers of interest: (A) itaconic acid, (B) citraconic acid, and (C) mesaconic acid. (D) TCA cycle diagram summarizing expression changes observed in activated BMDMs following 24 h of LPS exposure and the role of itaconic acid and its isomers in the inflammatory response. Relative abundances of the TCA intermediate (E) succinic acid and related metabolites (F) glutamic acid, (G) lactic acid, and (H) aspartic acid. *p < 0.05, **p < 0.001, ***p < 0.0001 for the LPS versus control comparison at that time point.

To demonstrate the quantitative performance within our untargeted metabolomics workflow, the unsaturated dicarboxylic acids itaconic, citraconic, and mesaconic acid were quantified, as they were of particular interest for this application (Figure S7). As anticipated, the measured concentration of itaconic acid significantly increased upon macrophage activation (Figure 4A, *p* = 8 × 10^–5^, FC = 2.2).^64,65^ This increase only occurred after 24 h of LPS stimulation, suggesting that at this concentration of LPS, 4 h was not enough time for itaconic acid accumulation to occur. The constitutional isomers of itaconic acid, citraconic and mesaconic acid, had concentration distributions reflecting that of itaconic acid, where expression of both citraconic acid (Figure 4B, *p* = 4 × 10^–6^, FC = 2.5) and mesaconic acid (Figure 4C, *p* = 0.02, FC = 2.2) significantly increased upon macrophage activation following 24 h of LPS exposure. The isomer concentrations ranged across 2 orders of magnitude, with citraconic acid > itaconic acid > mesaconic acid (Figure S8). The calculated concentrations are underestimations, as some partially derivatized products were detected at low abundance. This is not expected to impact interpretation since the complete and partially derivatized products shared the same abundance trends (Figure S6). The measured concentrations of all three isomers were higher in the 24 h unstimulated control BMDMs compared to the 4 h control BMDMs; however, these differences were not statistically significant (*p* > 0.05) and likely reflect ongoing steady state metabolism. Previous studies on metabolic reprogramming of LPS-activated macrophages have focused primarily on itaconic acid accumulation; however, two recent studies from He et al. and Chen et al. reported both itaconate and mesaconate accumulation with LPS stimulation of mouse macrophage RAW264.7 cells^66^ and LPS/interferon-γ (IFN-γ) stimulation of dTHP1 cells,^55^ respectively. He et al. did not attempt to measure citraconic acid, whereas Chen et al. reported citraconic acid as not detected in dTHP1 cells, regardless of activation, making this the first report of all three isomers increasing upon activation with LPS.

Induction of itaconic acid upon macrophage activation with LPS and other stimulating factors is well-studied and has been described in detail through several reviews.^64,65,67^ Briefly, stimulated macrophages experience increased expression of aconitate decarboxylase 1 (ACOD1), which diverts *cis*-aconitic acid away from the TCA cycle and converts it to itaconic acid (Figure 4D). Itaconic acid in turn competitively inhibits succinate dehydrogenase (SDH) activity, impacting the TCA cycle flux. Itaconic acid and its esterified derivatives have also been shown to regulate macrophage immune response through several mechanisms beyond inhibiting the TCA cycle.^64,65^ Less is understood about the role and molecular mechanisms of the endogenous isomers of itaconic acid (i.e., citraconate and mesaconate) in immune response; however, two recent studies have demonstrated their potential importance.^55,66^ Multiple works have demonstrated that mesaconic acid is synthesized intracellularly from itaconic acid.^55,66,68^ However, Chen et al. concluded that citraconic acid is not derived from itaconic acid or mesaconic acid^55^ and may be a derivative of isoleucine.^69^ Regardless of their biosynthesis, both citraconic and mesaconic acid appear to have immunomodulatory effects in macrophages, including a similar ability to alter the TCA cycle, although to a lesser extent than itaconate.^55^ Further studies are needed to discern the mechanisms of action of each isomer.

Untargeted metabolomics analysis revealed relative abundances of TCA cycle intermediates and related metabolites following the expected reprogramming of the TCA cycle following activation of macrophages and induction of itaconic acid (Figure 4D–H). The TCA cycle intermediates citric acid, α-ketoglutaric acid, fumaric acid, and malic acid were detected, but significant changes in expression upon activation were not observed (Figure 4D). Isocitric acid was not detected in the BMDM samples. Expression of succinic acid significantly increased upon macrophage activation by 24 h of LPS exposure (Figure 4E, *p* = 9 × 10^–4^, FC = 1.6), consistent with previous findings of succinic acid accumulation with itaconic acid competitively inhibiting SDH activity.^70,71^ No other changes in detected TCA cycle metabolites were observed; however, differences in the TCA products and substrates were differential. Glutamic acid abundance increased following 24 h of LPS exposure (Figure 4F, *p* = 0.002, FC = 3.2). Within the glutaminolysis pathway, glutamic acid is deaminated to the TCA cycle intermediate α-ketoglutaric acid, which then acts as an anaplerotic substrate in the TCA cycle.^72^ This route of replenishing the TCA cycle intermediates has been shown to be critical for the polarization of macrophages toward their anti-inflammatory phenotype.^73^ Interestingly, the abundance of lactic acid and aspartic acid was found to differ at both 4 and 24 h of LPS exposure. Lactic acid was upregulated at both time points (Figure 4G, *p* = 0.004 and 7 × 10^–5^, FC = 2.3 and 3.7 for 4 and 24 h, respectively), indicating the initial switch from oxidative phosphorylation to glycolysis to fuel the inflammatory response may occur before other TCA cycle intermediates undergo changes in expression.^74^ Decreased expression of aspartic acid was observed at both time points, with a more marked decrease at 4 h than 24 h (Figure 4H, *p* = 2 × 10^–4^ and 0.007, FC = −2.1 and −1.4 for 4 and 24 h, respectively). Aspartic acid depletion upon macrophage activation with LPS and interferon gamma has been demonstrated previously, with aspartic acid having its own roles in the inflammatory response including the promotion of interleukin-1β secretion and activation of inflammasomes.^75^

## Conclusions

LC-MS-based measurement of carboxyl-containing metabolites (CCMs) can be challenging as a result of CCM isomerism and their physiochemical properties, such as poor RPLC retention and ESI^–^ measurement. The addition of TIMS analysis can be useful for additional specificity and annotation confidence; however, researchers seeking comprehensive metabolomics data often turn to multiple injections with TIMS mode on and off to capture CCMs.^24,25^ Here, we introduce the carbodiimide-mediated coupling of 4-BNMA with CCMs to enhance their detection and recognition using typical untargeted LC-ESI-TIMS-MS/MS methods. Derivatization increased the signal of small CCMs such as pyruvic acid by several orders of magnitude and improved both RPLC retention and isomer separations. Moreover, 4-BNMA is an IMS shift reagent due to the presence of one or more bromine atoms disproportionally adding mass for a relatively smaller increase in size. Further, bromine provided unique isotope patterns used in data processing to improve specificity in data mining. We leveraged MassQL, a new data mining approach, to find signals in the raw data of putatively 4-BNMA derivatized CCMs. Shifting the mobility of derivatized CCMs aided in noise reduction, PASEF MS/MS selection, and classification of unknowns in addition to the unique isotope patterns. This workflow was applied to a proof-of-concept study evaluating the modulation of TCA cycle intermediates following LPS activation of bone marrow-derived macrophages. Enhanced separation of derivatized isomers allowed for analysis of a key immunomodulatory metabolite, itaconic acid, and its two endogenous isomers, citraconic and mesaconic acid, and the initial reporting of all three isomers increased upon LPS-activated BMDMs.

## Supporting Information Available

Summary of nonderivatized and derivatized standards; LC-IMS heatmap of derivatized itaconic acid isomers; energy-optimized derivatized structures; IMS-MS plot of derivatized standards; mobility filtering example; summary of CCMs detected in BMDM samples; partially derivatized CCM evaluation; calibration curves; and nonscaled isomer concentrations (PDF)
