# Supplementary material for: Bromine-Based Derivatization of Carboxyl-Containing Metabolites for Liquid Chromatography–Trapped Ion Mobility Spectrometry–Mass Spectrometry
Source: J Am Soc Mass Spectrom. 2025 Mar 7;36(4):888–99. doi: 10.1021/jasms.5c00023 (PMC11970421; doi:10.1021/jasms.5c00023)
Supplement: Supplementary file 1 — js5c00023_si_001.pdf [file js5c00023_si_001.pdf]

Supporting Information for

Bromine-Based Derivatization of Carboxyl-Containing Metabolites for Liquid Chromatography-Trapped Ion Mobility Spectrometry-Mass Spectrometry

Kaylie I. Kirkwood-Donelson[1], Prashant Rai[1], Lalith Perera[2], Michael B. Fessler[1], Alan K. Jarmusch[1]*

[1]Immunity, Inflammation, and Disease Laboratory, [2]Genome Integrity and Structural Biology Laboratory, National Institute of Environmental Health Sciences, National Institutes of Health, Research Triangle Park, NC 27709, USA

*Correspondence: alan.jarmusch@nih.gov

Table S1. Summary of LC-IMS-MS observations for nonderivatized and 4-BNMA derivatized carboxylic acid standards

Metabolite	Comparison	Formula	Adduct	*m/z*	RT (min)	CCS (Å²)
Pyruvic acid	Nonderivatized	$C_3H_4O_3$	$[M\text{-}H]^-$	87.008	0.6	110.3[2]
	4-BNMA derivatized	$C_{11}H_{13}NO_2Br$	$[M\text{+}H]^+$	270.012	5.4	152.8
Itaconic acid	Nonderivatized	$C_5H_6O_4$	$[M\text{-}H]^-$	129.019	0.8	120.3[2]
	4-BNMA derivatized	$C_{21}H_{23}N_2O_2Br_2$	$[M\text{+}H]^+$	493.012	9.3	199.9/207.0
Citraconic acid	Nonderivatized	$C_5H_6O_4$	$[M\text{-}H]^-$	129.019	0.7	120.4[2]
	4-BNMA derivatized	$C_{21}H_{23}N_2O_2Br_2$	$[M\text{+}H]^+$	493.012	9.0	195.2
Mesaconic acid	Nonderivatized	$C_5H_6O_4$	$[M\text{-}H]^-$	129.019	0.7	120.4[2]
	4-BNMA derivatized	$C_{21}H_{23}N_2O_2Br_2$	$[M\text{+}H]^+$	493.012	8.9	199.8
Citric acid	Nonderivatized	$C_6H_8O_7$	$[M\text{-}H]^-$	191.019	1.1	127.1[1]
	4-BNMA derivatized	$C_{30}H_{33}N_3O_4Br_3$	$[M\text{+}H]^+$	736.002	11.2	238.5
Isocitric acid	Nonderivatized	$C_6H_8O_7$	$[M\text{-}H]^-$	191.019	1.1	127.0[1]
	4-BNMA derivatized	$C_{30}H_{33}N_3O_4Br_3$	$[M\text{+}H]^+$	736.002	10.3	235.4

[1]Literature or [2]predicted CCS value

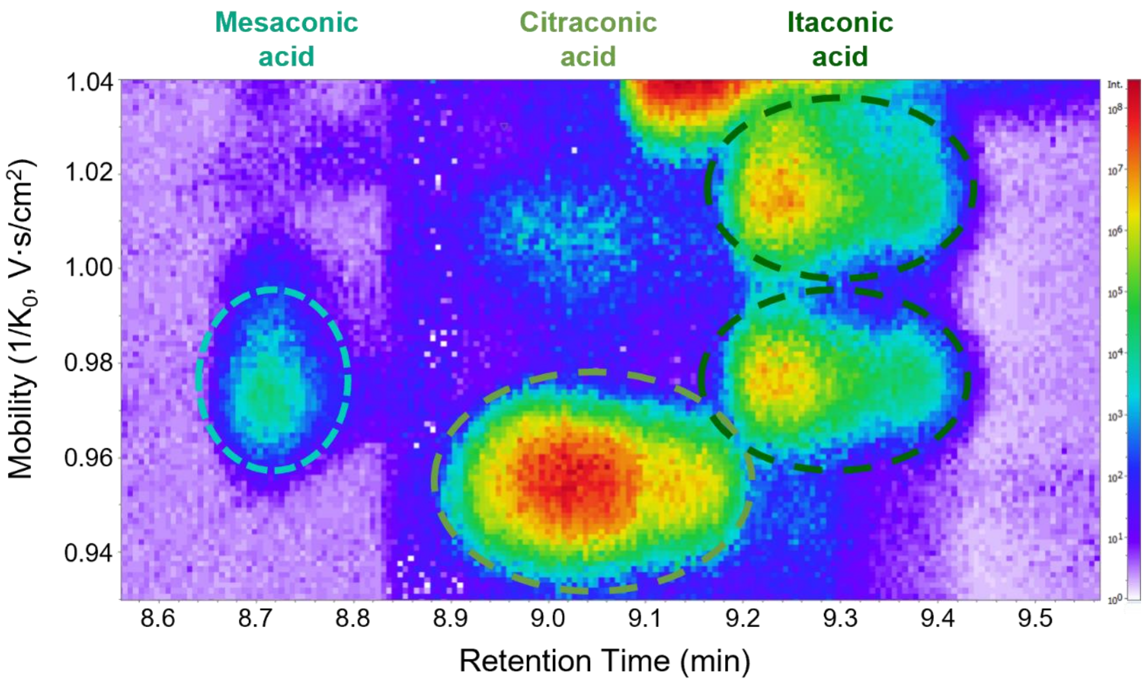

Figure S1. LC-IMS heatmap of 4-BNMA derivatized itaconic acid isomers. Zoomed heat map of mobility versus retention time showing one primary feature at the retention time of mesaconic acid (8.7 min), one primary feature at the retention time of citraconic acid (9.0 min) and two features at the retention time of itaconic acid (9.3 min), one of which is at the same mobility as mesaconic acid. The feature at the upper mobility limit of this heatmap (~1.04 V·s/cm^2) is an unrelated, non-brominated feature (*m/z* 394.379) with a retention time of 9.2 min.

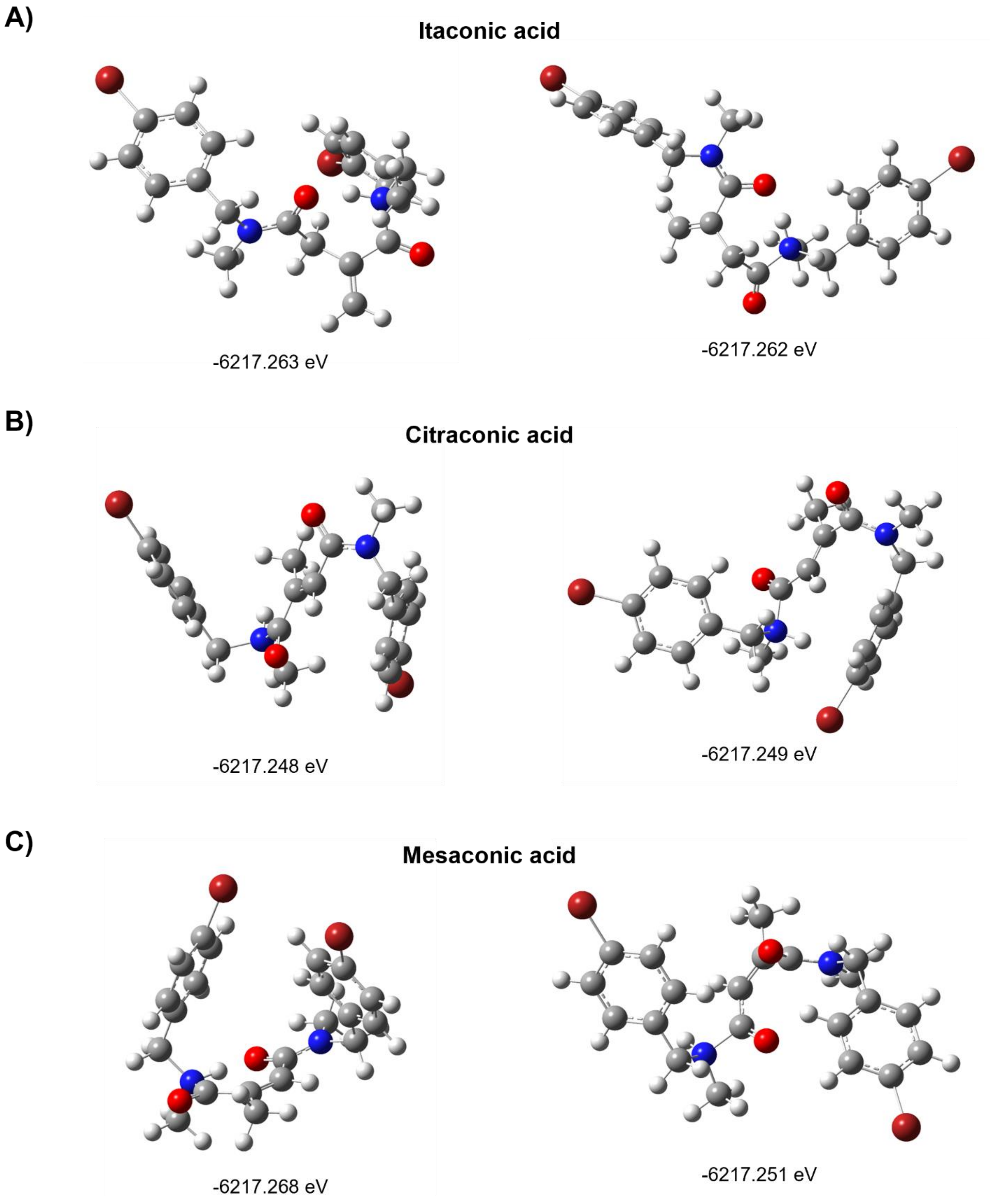

Figure S2. Energy-optimized isomer structures. The top minimum energy conformation is shown for both possible protonation sites for **A**) itaconic acid, **B**) citraconic acid, and **C**) mesaconic acid.

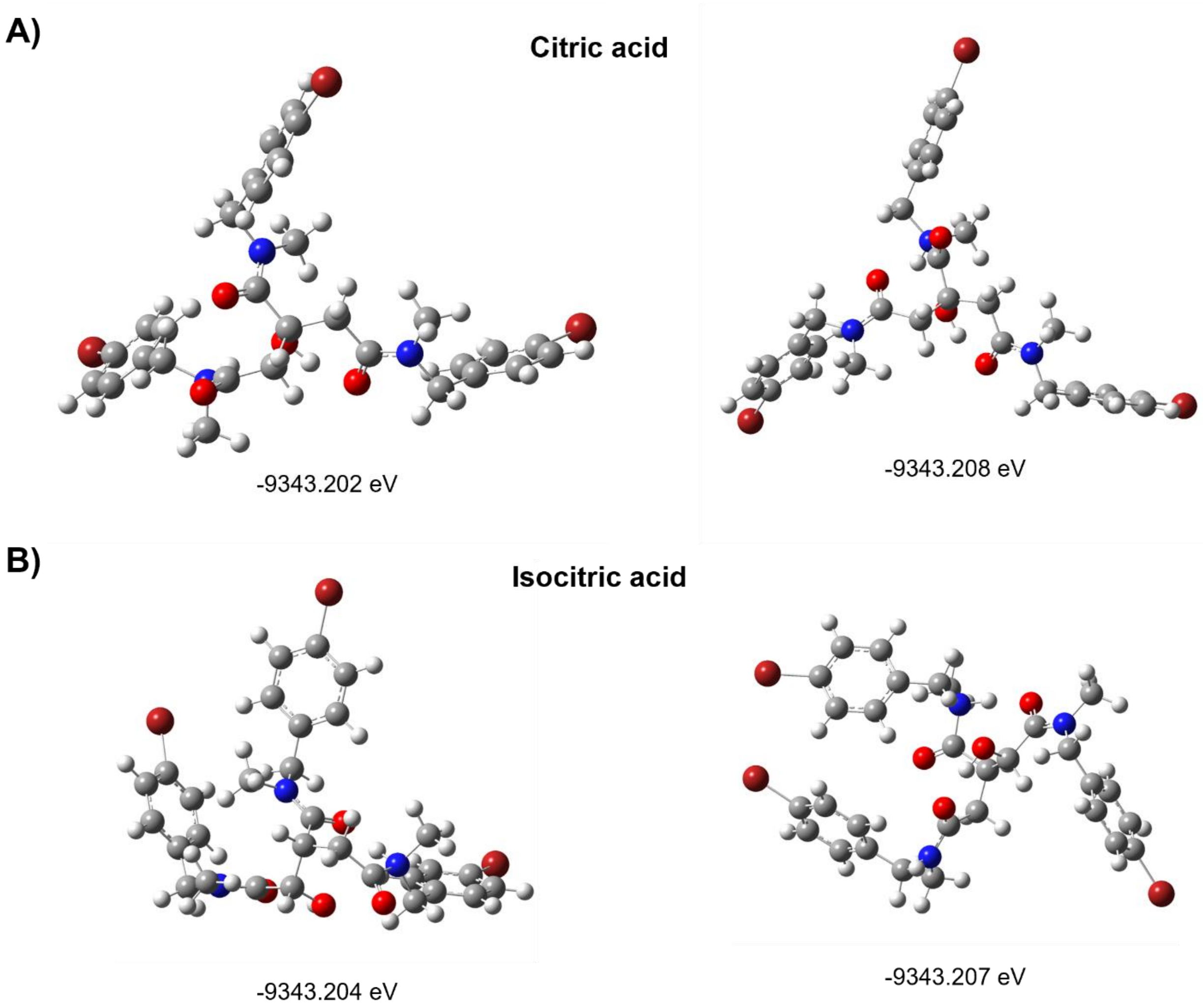

Figure S3. Energy-optimized isomer structures. The top minimum energy conformation is shown for the two asymmetric protonation sites for **A**) citric acid and **B**) isocitric acid.

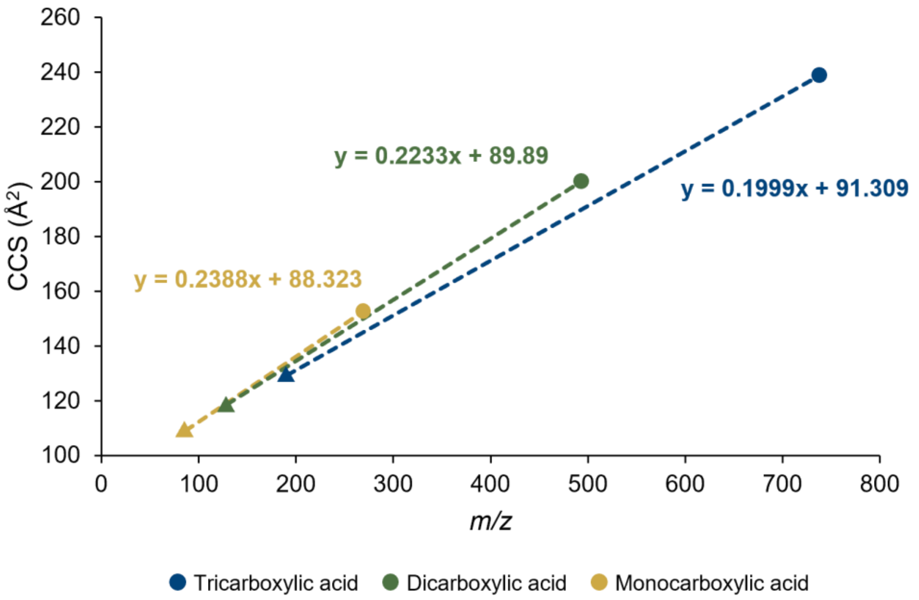

Figure S4. IMS-MS plot of derivatized carboxylic acid standards. Lines were generated using the CCS and *m/z* values for representative nonderivatized (triangle)/derivatized (circle) pairs of monocarboxylic acid (pyruvic acid), dicarboxylic acid (itaconic acid), and tricarboxylic acid (citric acid) standards.

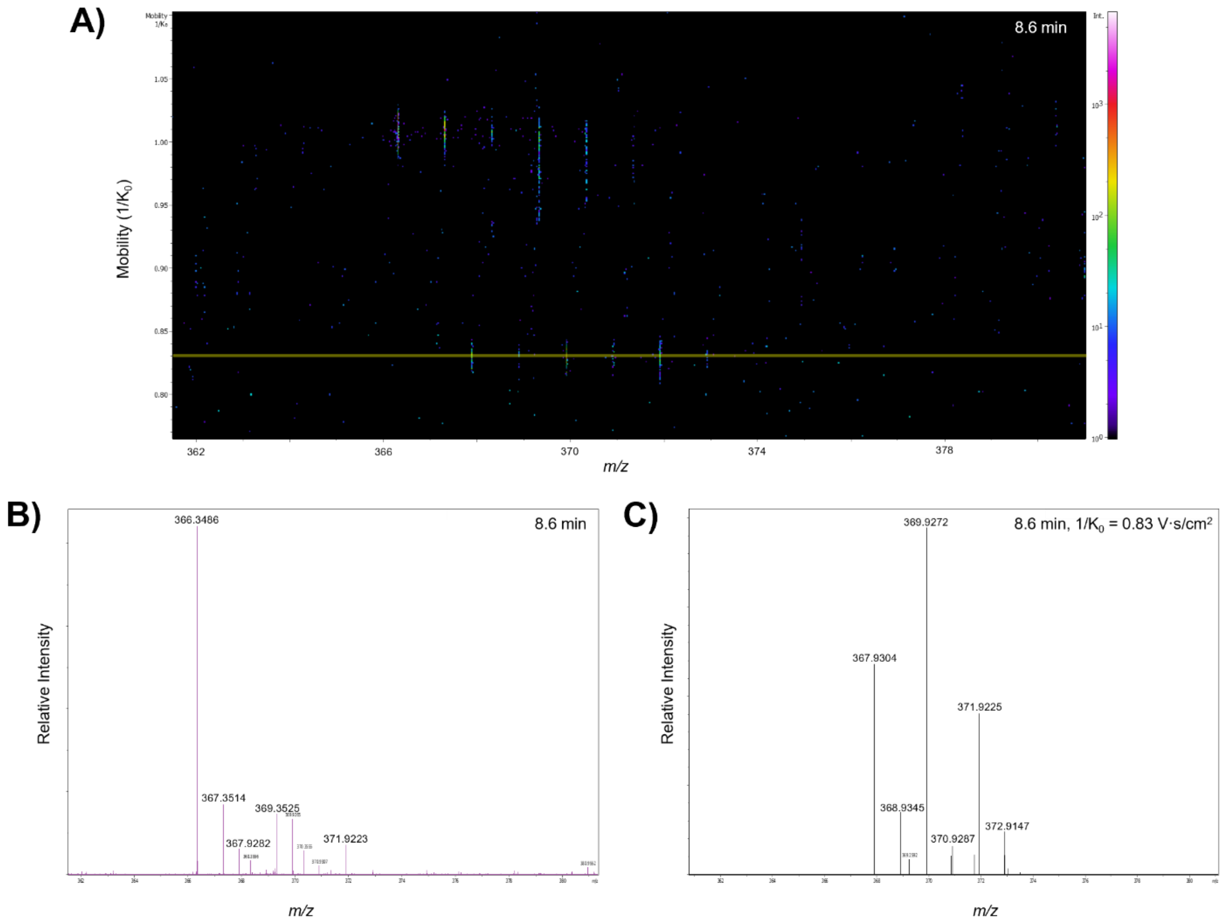

Figure S5. Noise reduction with mobility filtering in BMDM samples. A) Zoomed IMS-MS heatmap at 8.6 min showing features with overlapping isotopic distributions, at inverse reduced mobilities of 1.01 and 0.83 V·s/cm^2. Comparison of zoomed MS spectra **B**) before mobility filtering and **C**) after imposing a filter for signal at an inverse reduced mobility of 0.83 V·s/cm^2, shown as a yellow line in **A**, revealing a dibrominated ion which may be overlooked if IMS was not used.

Table S2. Summary of LC-IMS-MS observations for all 50 unique carboxylic acids detected in BMDM samples.

# Br	*m/z*	RT (min)	CCS (Å²)	Known ID	Tentative ID or Formula
1	270.012	5.4	152.8	Pyruvic acid	-
1	256.033	6.9	151.6	-	Propionic acid
1	272.028	5.7	154.9	-	Lactic acid
1	311.039	5.1	168.1	-	Pyroglutamic acid
1	313.055	5.2	165.8	-	Hydroxyproline
1	378.055	3.8	180.2	-	Gluconic acid
1	385.112	3.9	185.3	-	Acetylcarnitine
1	293.991	4.6	152.7	-	-
1	305.005	5.7	157.1	-	C6H4O3
1	323.086	5.2	170.1	-	-
1	333.021	5.1	175.5	-	-
1	337.055	5.2	164.37	-	-
1	337.055	5.9	173.1	-	-
1	347.036	5.4	168.6	-	-
1	411.177	4.5	191.8	-	-
1	419.019	4.9	193.8	-	-
1	435.116	4.8	186.8	-	C12H18N2O4
1	437.156	4.7	191.1	-	C14H24O4
1	449.155	4.8	194.0	-	-
1	465.186	5.0	198.3	-	-
1	467.165	4.8	195.0	-	-
1	468.059	7.5	202.6	-	-
1	483.161	5.1	201.2	-	-
1	528.113	5.3	209.1	-	-
1	532.168	5.3	211.4	-	-
1	555.010	8.0	216.5	-	-
1	555.010	8.6	214.4	-	-
2	493.012	9.3	207.0	Itaconic acid	-
2	493.012	9.0	195.2	Citraconic acid	-
2	493.012	8.9	199.8	Mesaconic acid	-
2	460.963	7.2	182.2	-	Phosphate
2	478.996	8.6	197.8	-	Fumaric acid
2	481.012	8.9	196.9	-	Succinic acid
2	495.028	9.4	199.7	-	Glutaric acid
2	496.023	5.7	196.9	-	Aspartic acid
2	497.007	8.2	197.2	-	Malic acid
2	510.039	9.0	200.3	-	Glutamic acid
2	538.035	7.8	205.9	-	N-acetylaspartic acid
2	639.120	6.0	227.8	-	Saccharopine*

2	536.056	5.7	201.5	-	-
2	568.042	8.4	211.3	-	-
2	616.105	5.2	223.8	-	-
2	616.105	5.5	224.7	-	-
2	696.071	5.3	240.2	-	-
3	736.002	11.2	238.5	Citric acid	-
3	736.002	10.3	235.4	Isocitric acid	-
3	704.014	7.3	235.5	-	-
3	704.014	7.9	234.6	-	-
3	710.161	6.4	237.9	-	-
3	710.161	6.6	237.8	-	-

*Partially derivatized (m/z for complete derivatization not detected)

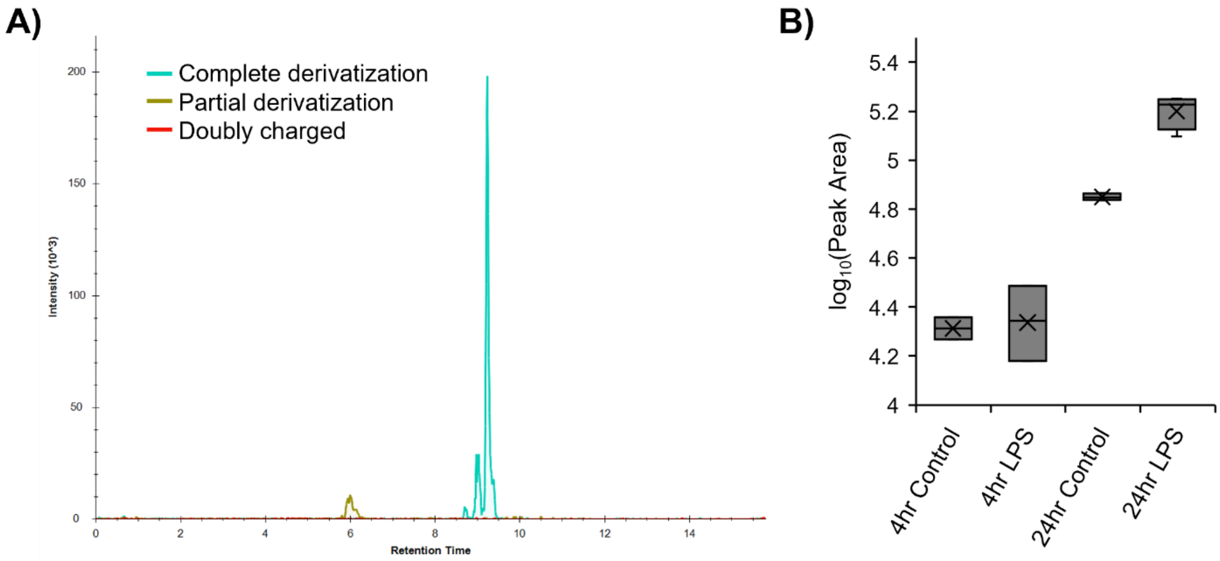

Figure S6. **Evaluation of partially derivatized products in complex matrix. A**) EICs for complete derivatization (blue, *m/z* 493.012), partial derivatization (yellow, *m/z* 312.0214), and doubly charged (red, *m/z* 248.018, not detected) itaconic, citraconic and mesaconic acid isomers. **B**) Log_{10}-transformed total peak area for the partially derivatized itaconic acid isomers demonstrating similar differences across sample groups as the fully derivatized itaconic acid isomers (**Figure 4A-C**).

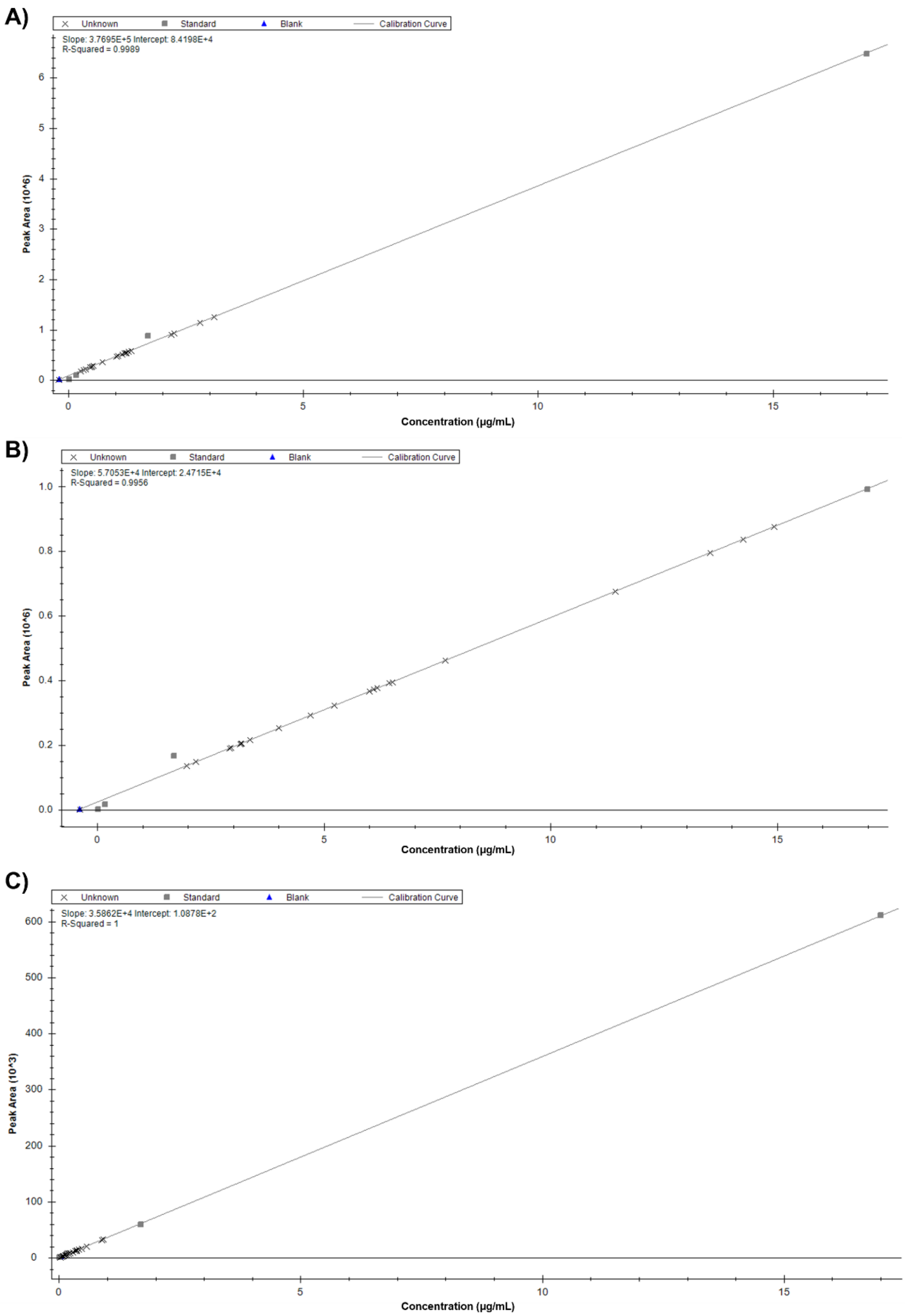

Figure S7. Calibration curves. External calibration curves for quantitation of **A**) itaconic acid, **B**) citraconic acid and **C**) mesaconic acid.

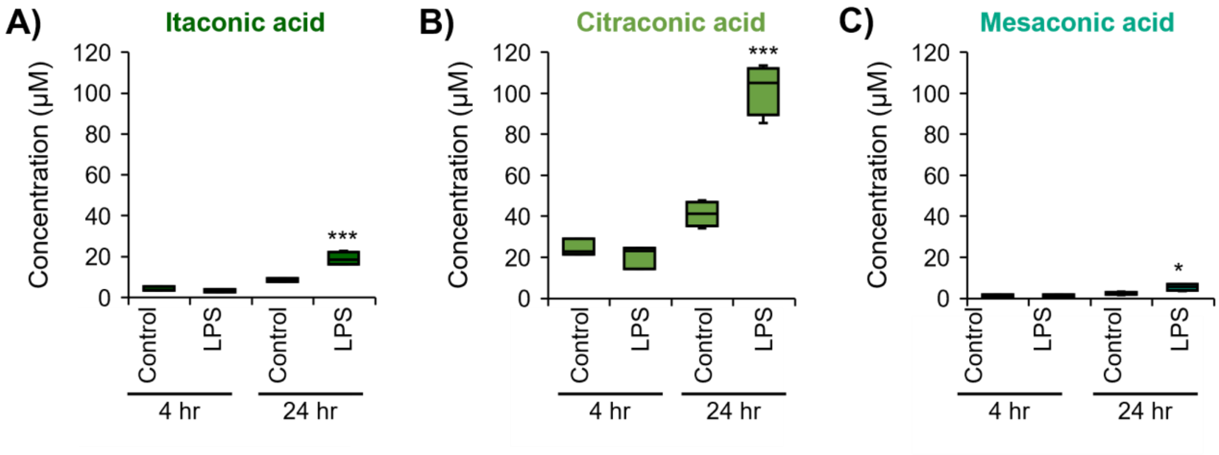

Figure S8. Non-scaled concentrations. Calculated concentrations of the isomers of interest, **A**) itaconic acid, **B**) citraconic acid, and **C**) mesaconic acid plotted on the same concentration scale.

References

1. Nichols, C. M.; Dodds, J. N.; Rose, B. S.; Picache, J. A.; Morris, C. B.; Codreanu, S. G.; May, J. C.; Sherrod, S. D.; McLean, J. A., Untargeted Molecular Discovery in Primary Metabolism: Collision Cross Section as a Molecular Descriptor in Ion Mobility-Mass Spectrometry. *Analytical Chemistry* **2018**, *90* (24), 14484-14492.
2. Ross, D. H.; Cho, J. H.; Xu, L., Breaking Down Structural Diversity for Comprehensive Prediction of Ion-Neutral Collision Cross Sections. *Anal Chem* **2020**, *92* (6), 4548-4557.
